# Proceedings of the 6th Asia Dengue Summit, June 2023

**DOI:** 10.1371/journal.pntd.0012060

**Published:** 2024-03-29

**Authors:** Nattachai Srisawat, Duane J. Gubler, Tikki Pangestu, Umaporn Limothai, Usa Thisyakorn, Zulkifli Ismail, Daniel Goh, Maria Rosario Capeding, Lulu Bravo, Sutee Yoksan, Terapong Tantawichien, Sri Rezeki Hadinegoro, Kamran Rafiq, Valentina Sanchez Picot, Eng Eong Ooi

**Affiliations:** 1 Tropical Medicine Cluster, Center of Excellence in Critical Care Nephrology, Faculty of Medicine, Chulalongkorn University, Excellence Center for Critical Care Nephrology, King Chulalongkorn Memorial Hospital, Thailand; 2 Program in Emerging Infectious Diseases, Duke-NUS Medical School, Singapore; 3 Yong Loo Lin School of Medicine, National University of Singapore, Singapore; 4 Tropical Medicine Cluster, Chulalongkorn University and Faculty of Tropical Medicine, Mahidol University, Bangkok, Thailand; 5 Department of Pediatrics, KPJ Selangor Specialist Hospital, Malaysia; 6 Division of Paediatric Pulmonary Medicine and Sleep, Khoo Teck Puat National University Children’s Medical Institute, National University Hospital, Singapore; 7 Research Institute for Tropical Medicine, Muntinlupa City, Manila Metro, Philippines; 8 University of the Philippines Manila, Manila, the Philippines; 9 Center for Vaccine Development, Institute of Molecular Biosciences, Mahidol University, Bangkok, Thailand; 10 Division of Infectious Diseases, Department of Medicine and Tropical Medicine Cluster, Chulalongkorn University, Bangkok, Thailand; 11 Department of Child Health, Faculty of Medicine Universitas Indonesia, Jakarta, Indonesia; 12 International Society of Neglected Tropical Diseases, London, United Kingdom; 13 Fondation Merieux, Lyon, France; 14 Program in Emerging Infectious Diseases, Duke-NUS Medical School, Singapore; University of Calgary, CANADA

## Abstract

The 6th Asia Dengue Summit (ADS) themed “Road Map to Zero Dengue Death” was held in Thailand from 15th–16th June 2023. The summit was hosted by Tropical Medicine Cluster, Chulalongkorn University, Bangkok, Thailand in conjunction with Queen Saovabha Memorial Institute, The Thai Red Cross Society; Faculty of Tropical Medicine, Mahidol University; and the Ministry of Public Health. The 6th ADS was convened by Asia Dengue Voice and Action (ADVA); Global Dengue and Aedes Transmitted Diseases Consortium (GDAC); Southeast Asian Ministers of Education Tropical Medicine and Public Health Network (SEAMEO TROPMED); Fondation Mérieux (FMx) and the International Society for Neglected Tropical Diseases (ISNTD). Dengue experts from academia and research, and representatives from the Ministries of Health, Regional and Global World Health Organization (WHO) and International Vaccine Institute (IVI) participated in the three-day summit. With more than 51 speakers and 451 delegates from over 24 countries, 10 symposiums, and 2 full days, the 6^th^ ADS highlighted the growing threat of dengue and its antigenic evolution, flagged the urgent need to overcome vaccine hesitancy and misinformation crisis, and focused on dengue control policies, newer diagnostics, therapeutics and vaccines, travel-associated dengue, and strategies to improve community involvement.

## 1. Introduction

The 6th Asia Dengue Summit (6^th^ ADS) was held in Thailand from 15th–16th June 2023. The 6th ADS was hosted by Tropical Medicine Cluster, Chulalongkorn University, Bangkok, Thailand in conjunction with Queen Saovabha Memorial Institute, The Thai Red Cross Society; Faculty of Tropical Medicine, Mahidol University; and the Ministry of Public Health. The 6^th^ ADS was convened by Asia Dengue Voice and Action (ADVA); Global Dengue and Aedes Transmitted Diseases Consortium (GDAC); Southeast Asian Ministers of Education Tropical Medicine and Public Health Network (SEAMEO TROPMED); Fondation Mérieux (FMx) and the International Society for Neglected Tropical Diseases (ISNTD).

With 51 speakers and 451 delegates from over 24 countries, 10 symposiums, and 2 full days, the 6th ADS highlighted strategies to conquer the growing threat of dengue through the theme “Road Map to Zero Dengue Death”. The theme coincided with the World Health Organization (WHO) target to reduce preventable dengue deaths to zero by 2030. The summit included topics ranging from evolving dengue epidemiology, dengue in elderly and those with co-morbidities, organopathy and severe dengue, and virology and immunology, to environmental influence on spread of dengue, strategies for vector control, and newer developments in vaccines and therapeutics.

The summit highlighted key challenges from shifting dengue epidemiology, dual infection with dengue and COVID-19, vaccine hesitancy and misinformation crisis. However, the focus was on solutions through newer diagnostics and therapeutics, newer vaccines and technological advances, and strategies to improve community involvement. The summit provided an ideal opportunity for healthcare care professionals, researchers, epidemiologists, and representatives from the Ministries of Health to share strategies for successful dengue control. Keeping in line with its theme “Road Map to Zero Dengue Death”, the summit emphasized key approaches for dengue control–strengthening epidemiological surveillance. improving clinical awareness and preparedness, continued innovations in dengue diagnostics and therapeutics, vector control, vaccine advocacy, and multi-sectoral collaboration. This report summarizes the key highlights from the speaker presentations during the 6th Asia Dengue Summit.

## 2. Growing Threat of Dengue

Dengue poses an enormous threat to global public health with unprecedented human and economic cost, which is likely to be even higher than estimated. Dengue is endemic in over 128 countries with 3.6 billion people living in high-risk areas resulting in 390 million infections per year. Asia bears 70% of the global dengue burden particularly affecting people under 14 or over 70 years of age [1]. With growing population and urbanization the risk of dengue in Southeast Asia is predicted to rise even more warranting the need for robust and sustained dengue control efforts. Furthermore, with the added challenges from global warming and increased international travel, the risk of dengue spread to non-endemic countries poses a global health security risk [2].

With the coronavirus pandemic, the Southeast Asian nations faced a dual challenge with the already existing socio-economic burden of dengue. Co-infection with dengue and coronavirus, similar symptomatology, and cross-reactivity to rapid diagnostic tests increased the risk of missed diagnosis or delayed diagnosis further impairing urgent management and isolation procedures [3]. A recent study from Thailand indicated that the number of reported dengue cases declined between 2019 and 2021, corresponding to higher numbers of COVID-19 patients from the delta strain. This discovery raises the possibility that the government’s implementation of social distancing measures during the COVID-19 pandemic might have reduced dengue virus transmission [4]. However, lockdown policies hindered full implementation of dengue control and surveillance measures, highlighting the critical need for sustained dengue preparedness through continued vector control and strengthening of surveillance infrastructure. Digitization of dengue surveillance systems (e.g., digitization of house-to-house inspections, mobile-based surveillance) is one of the suggested strategies to strengthen remote dengue surveillance and vector control in Southeast Asia [3].

## 3. Improving Clinical Vigilance

There have been significant improvements in dengue clinical outcomes through advanced dengue case management, however there is still much more to be done. Strengthening of healthcare infrastructure, diagnostic and therapeutic capacity building, and multi-disciplinary coordination are critical aspects for improving clinical outcomes. Maintaining a high level of clinical vigilance is crucial, especially in select high-risk populations. A trend of shift in age groups towards older age groups has been seen during the past decade [5–7]. Presence of non-communicable comorbidities is known to increase the risk of severe dengue and complications. Individuals with underlying co-morbidities such as cardiovascular disease, stroke, diabetes, respiratory disease and renal disease, and old age are at an increased risk of developing severe disease and dengue related complications [8]. Studies in Mexico, Brazil, and Colombia show increased case fatality rate in hospitalized patients with underlying co-morbidities [9].

The prevalence of overweight and obesity among children worldwide is steadily increasing. Recent meta-analysis findings suggest that individuals with obesity face a 50% higher risk of developing severe dengue manifestations [10]. In a recent retrospective study involving 858 Vietnamese children with dengue, researchers observed a higher incidence of decompensated Dengue Shock Syndrome (DSS) in the obese cohort compared to the non-obese group. Importantly, obesity was significantly associated with the risk of severe respiratory failure requiring mechanical ventilation support [11]. Therefore, it is essential to closely monitor obese children with DSS for signs of severe respiratory distress and the need for high-flow oxygenation, particularly mechanical ventilation support, soon after hospitalization.

Higher mortality in dengue patients in intensive care units (ICU) is associated with lower Glasgow Coma Scale (GCS) scores, lower platelet counts, and organ failures [12]. Timely management of plasma leakage, severe bleeding, and/or organ impairment is crucial in such cases. Judicious fluid administration under clinical monitoring and serial hematocrit values constitutes the foundation of management of critical phase. Crystalloids are initial choice of fluids in dengue shock syndrome while prophylactic platelet transfusion is not recommended [13].

Pregnant women with dengue infection are considered at-risk population because of the increased risk of stillbirth and neonatal mortality, dengue shock syndrome and maternal mortality [14]. Vertical transmission of dengue from the mother to the neonate is more likely if maternal dengue infection occurs later during the pregnancy and closer to the delivery. In the event of known maternal dengue infection or fever 15 days before delivery, cord blood and placenta should be sampled and analyzed for neonatal dengue infection. In such cases, the newborn needs to be closely monitored during the post-partum period [15].

## 4. Antigenic Evolution of Dengue Viruses

Antigenic evolution of dengue viruses (DENV) is a highly complex process enabling evasion of host immune response, modifying future disease risk and affecting vaccine efficacy. Primary infection or vaccination induces homotypic immunity, which protects against secondary infection with the same serotype, however because each serotype comprises of different genotypes, protection is not always complete. During secondary infection with a different serotype, high levels of preexisting heterotypic antibodies can protect against infection, nonetheless low to intermediate levels increase the risk of severe disease through Antibody Dependent Enhancement (ADE). A recent study evaluating antigenic evolution of dengue viruses in Bangkok, Thailand between 1994–2014 showed antigenic changes within and among all serotypes resulting in an overall 40% reduction in neutralization over 20 years, thus influencing the magnitude of epidemics during that period [16]. This antigenic evolution is mediated through multiple mechanisms such as immune evasion, ADE, constraints on viral protein structure and introduction of new genotype. Evasion of homotypic or heterotypic immunity from the previous outbreak promotes strains to evolve with more antigenic difference from the earlier strains to drive the subsequent epidemic. Alternatively, if the level of preexisting immunity from the previous epidemic is low enough to mediate ADE, it may pull a serotype antigenically towards other serotypes to increase viral load and orchestrate the next epidemic. Then again, the need for DENV fitness in human host and mosquito vector may impose limitations on the viral protein structure and limit antigenic change, while genotype replacement may facilitate rebound increase in incidence [16]. Advancing understanding of these complex mechanisms of antigenic evolution is crucial for dengue surveillance and vaccine development.

## 5. Dengue Vaccine Update

Disease prevention and control is a key to public health. Dengue vaccine quest has utilized distinct tactics including live attenuated vaccines, inactivated whole-virus vaccines, vaccine-like particles, and DNA-based approaches. Live attenuated tetravalent formulations produced by Sanofi Pasteur, Takeda, and National Institutes of Health (NIH)/Butantan have been the leading dengue vaccine candidates. Takeda and NIH/Butantan use dengue virus (DENV) itself as a backbone, while flavivirus Yellow Fever forms the backbone in the Sanofi vaccine.

Dengvaxia is the only commercially licensed dengue vaccine for children aged 9–16 years in 20 countries. Though Dengvaxia demonstrated vaccine efficacy of 65.6 % and 44.6 % in children over 9 years and those younger than 9 years of age respectively, there were two main drawbacks. Dengvaxia offered lower protection in children under 9 years of age and did not develop heterotypic neutralizing antibodies against all four DENV serotypes. The efficacy of Dengvaxia was 74.0% against DENV3, 77.4% against DENV4, and 50.3% efficacy against DENV1 and 42.3% against DENV2. With a predominant immune response against DENV4 and a lower protection against DENV 1 and DENV 2, Dengvaxia functioned as a monovalent vaccine against DENV4 [17].

Furthermore, a safety signal raised during the third year of the Dengvaxia phase III clinical trial, highlighted a major setback with increased risk of hospitalization and severe dengue in the youngest non-immune vaccinated individuals [18,19]. Imbalanced immunity against all four dengue serotypes, antibody-dependent enhancement (ADE) in seronegative individuals, absence of DENV non-structural proteins in the vaccine construct and young age were the postulated mechanisms for increased risk of severe disease in seronegative vaccinated individuals [20]. Consequently, World Health Organization Strategic Advisory Group of Experts on Immunization (SAGE) revised its recommendation on Dengvaxia for use only in dengue immune individuals [21].

The other two dengue vaccine candidates, TAK-003 and NIH/Butantan/Merck vaccine have shown positive development. Indonesia, European Commission, and Brazilian regulators have recently approved Takeda vaccine (TAK-003) for use in individuals 4 years of age and older, irrespective of baseline dengue immune status [20]. In the phase III trial TAK-003 demonstrated a vaccine efficacy of 76.1% in dengue immune recipients and 66.2% in dengue non-immune recipients at the end of 18 months. TAK-003 established a vaccine efficacy of 90.4% against hospitalized dengue and 85.9% against dengue hemorrhagic fever [22]. The publication of data from the third vaccine candidate from the Instituto Butantan, U.S. NIH, and Merck (MSD) phase III trial in Brazil with over 16,000 participants is likely to be available soon.

## 6. Overcoming Dengue Vaccine Hesitancy

The Vaccine Confidence Project demonstrated that vaccine confidence tumbled from 93% of public agreeing that vaccines are important in 2015 to only 32% in 2018 [23]. It highlighted the crucial need to identify breaks in public confidence and restore trust in safety and efficacy of vaccines.

Vaccination is among the most cost-effective methods of disease prevention. However, vaccine hesitancy in common and is among the top 10 global health threats [24]. Misinformation about vaccines and negative healthcare experience, lack of trust in authorities [25] and inconvenience in vaccine access are the main contributors to vaccine hesitancy [24]. Public education and continuous vaccine advocacy by medical community, policy makers and media are critical in improving vaccine uptake. The WHO guide to Tailoring Immunization Programs (TIP) provides tools to identify vaccine hesitancy in subgroups of population and develop targeted strategies to improve vaccine uptake. The TIP has been successfully implemented in European countries like Bulgaria, Sweden and United Kingdom to improve vaccine hesitancy. The SAGE Working Group on Vaccine Hesitancy indicates that wider application of TIP can serve as a useful instrument to deal with vaccine hesitancy in other parts of the world [26].

Front-line healthcare providers are crucial in maintaining the public confidence in vaccines. In most countries’ physicians, and particularly general practitioners play a key role in vaccination and their recommendations impact patients’ vaccine approach. However, vaccine hesitancy among healthcare providers (HCPs) is a major barrier to successful implementation of vaccination programs. Vaccine hesitancy among general practitioners results from doubts about vaccine safety and distrust in health authorities. Educating HCPs on the risks and benefits of vaccination and training them on effective communication with vaccine-hesitant patients is crucial to overcome vaccine hesitancy [27].

## 7. Innovations in Vector Control

Traditional vector control approaches such as larval source reduction and adulticide interventions are not sufficient for effective dengue control mainly due to lack of sustainable strategies and insecticide resistance. The WHO Vector Control Advisory Group (WHO VCAG) is continually evaluating several innovative vector strategies. Use of spatial repellents (SR) is one such strategy, which releases volatile active ingredients in the air to prevent mosquitoes from entering sprayed spaces, interfering with their ability to bite humans and affecting their reproductive performance. Clinical trials from Indonesia and Peru demonstrate the capability of metofluthrin-based and transfluthrin-based SR in reducing the incidence of malaria and Aedes viral infections, however further evidence is required to establish their efficacy for public health use [28]. Attractive targeted sugar bait (ATSB) technology using sugar baits with toxins to attract mosquitoes for a feed is another emerging vector control strategy with promising results and potential for incorporation in the integrated vector management [29]. Use of permethrin impregnated school uniforms is one more novel strategy that has demonstrated significant reduction in the number of Aedes mosquitoes in a Thai study, however the protective effect did not translate in reduced number of dengue infections, presumably due to wash-out of permethrin during laundering [30]. Further research on long-lasting insecticide-treated clothing materials that can withstand washing is necessary.

## 8. Role of Asia Dengue Voice and Action (ADVA)

Dedicated to dengue prevention and control, Asia Dengue Voice and Action (ADVA), is a scientific working group established in 2011. ADVA’s mission is to strengthen dengue preparedness and develop a regional blueprint for dengue management through continuous educational programs and vaccine advocacy. ADVA acknowledges the role of international expert discussion and experience exchange in management of complex dengue cases and reducing case fatality rate. Consistent with this principle of international collaboration and resource mobilization, ADVA holds regular webinar series on dengue management to generate well-informed, focused, and coordinated actions across Asia.

Through collaboration with academia, industry and non-government organizations, ADVA has continued its dengue control efforts during the coronavirus pandemic through numerous local and regional workshops and webinars. To raise awareness on dengue, ADVA supports ASEAN Dengue Day on June 15 and holds several educational initiatives every year. ADVA and the International Society for Neglected Tropical Diseases (ISNTD) jointly hosted the ISNTD-ADVA World Dengue Day Forum in June 2021 and announced a strong call to action to establish World Dengue Day to improve worldwide responsiveness and preparedness against dengue through international collaboration, resource mobilization and shared responsibility [31].

The ADVA Dengue Task Force was set up in June 2022 with the objective to strengthen dengue preparedness and responsiveness in Asia and is continuing its efforts through identification of gaps in dengue control, continuous research, building strong dengue database, vaccine advocacy and communication with policy makers. The 6th ADS initiated the Young ADVA program by engaging youth in dengue prevention and control to strengthen the sustainability of overall dengue control mission. The presentations by young ADVA representatives and discussions gathered critical information on development of simple, easy to understand and impactful strategies (e.g., Using social media platforms, engaging youth influencers) to facilitate dengue education among the community, particularly among younger population.

## 9. Dengue Infections in Travellers

Greater connectivity across the world and increased international travel threatens the spread of dengue to non-endemic regions. Dengue infection is one of the most common causes of fever in travellers returning from dengue endemic regions. Dengue infected travellers put the local population at risk of infection and increase the risk of spread of dengue to non-endemic regions. In a recent prospective European multicentre cohort study 40% of returning travellers presenting with acute undifferentiated febrile illnesses were identified as malaria or dengue [32]. Highest travellers’ infection rate (TIR) of 6.1 cases /100,000 travellers has been reported in European travellers returning from Asia from 2015 to 2019 [33].

United States of America reported a 168% upsurge in travel-associated dengue cases in 2019 compared to the annual average during 2010–2018 and 2020–2021. Travel related dengue cases were associated with travel to Caribbean (39%), Asia (27%) and North America (14%) with most cases occurring during July–November [34]. Travel-associated dengue imposes significant implications for public health practice. Healthcare providers in non-endemic regions need to be familiar with travel associated dengue and institute appropriate diagnostic testing and management. There is a need to establish robust surveillance systems to monitor dengue infections in returning travellers. Travelers should be educated on risk of dengue in endemic regions, follow guidelines to avoid mosquito bites and seek prompt medical attention in case of fever after returning home.

## 10. Conclusion

**Fig 1:**
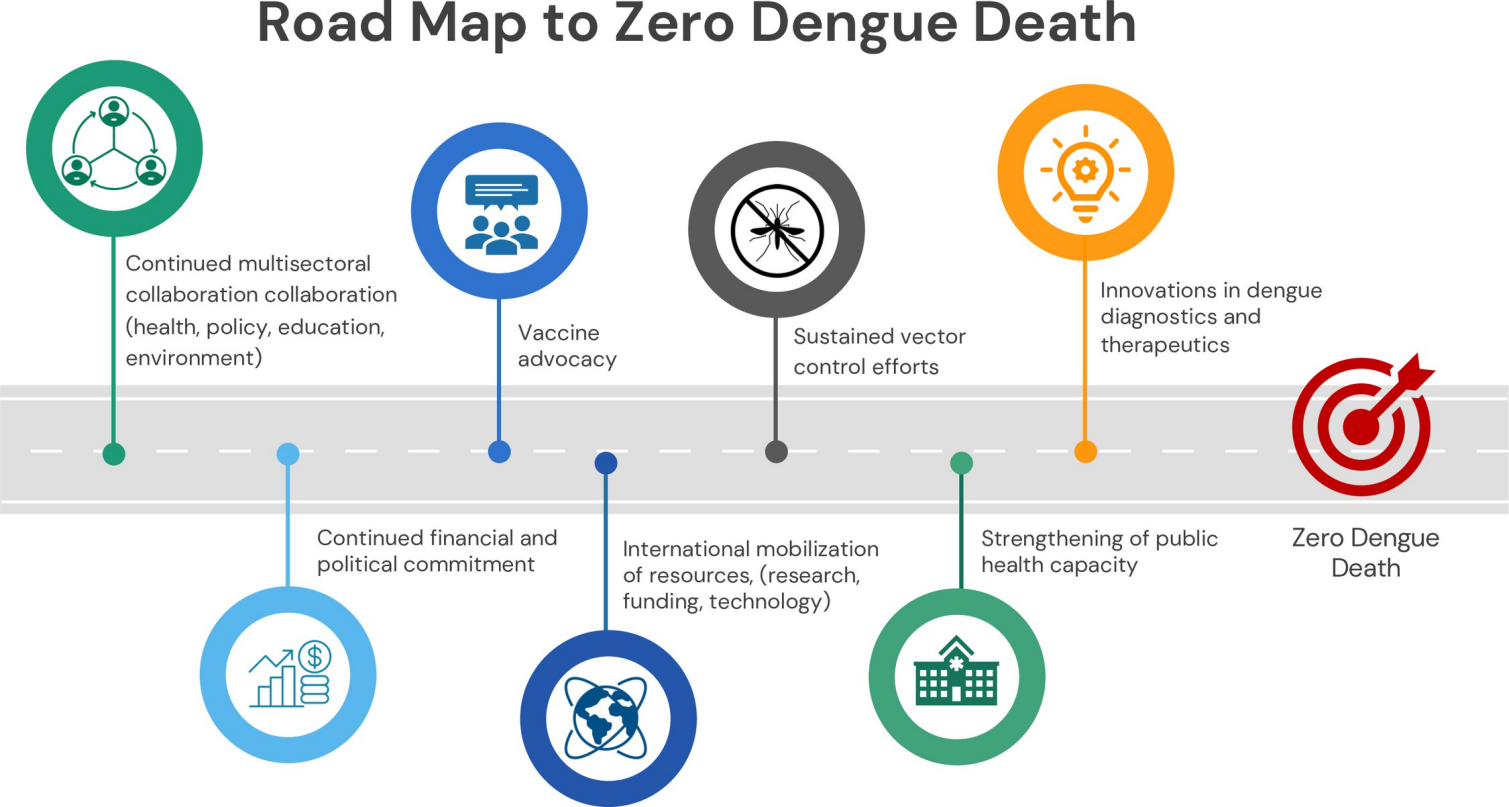
Road Map to Zero Dengue Death.

There is no single panacea for dengue control. Continued multisectoral collaboration (health, policy, education, environment), international mobilization of resources, (research, funding, technology) continued financial and political commitment, strengthening of public health capacity, and sustained vector control efforts, innovations in dengue diagnostics and therapeutics, and vaccine advocacy are imperative to achieve the target of zero dengue deaths (Fig 1). With continuous and collective effort and newer tools in the pipeline, the world is entering a new era that might bring us closer to rollback dengue. Residual insecticides, genetic control, biomarkers for severe disease, therapeutic antibodies, antiviral drugs, and vaccines show promising potential to tackle dengue.
